# Impact of Protein Aggregates on Sporulation and Germination of *Bacillus subtilis*

**DOI:** 10.3390/microorganisms11092365

**Published:** 2023-09-21

**Authors:** Julien Mortier, Alexander Cambré, Sina Schack, Graham Christie, Abram Aertsen

**Affiliations:** 1 Department of Microbial and Molecular Systems, KU Leuven, B-3000 Leuven, Belgium; julien.mortier@vib.be (J.M.); alexander.cambre@kuleuven.be (A.C.); 2 Department of Chemical Engineering & Biotechnology, University of Cambridge, Cambridge CB3 0AS, UK; sina.schack@outlook.com (S.S.); gc301@cam.ac.uk (G.C.)

**Keywords:** protein aggregate, *Bacillus subtilis*, sporulation, germination

## Abstract

In order to improve our general understanding of protein aggregate (PA) management and impact in bacteria, different model systems and processes need to be investigated. As such, we developed an inducible synthetic PA model system to investigate PA dynamics in the Gram-positive model organism *Bacillus subtilis*. This confirmed previous observations that PA segregation in this organism seems to follow the *Escherichia coli* paradigm of nucleoid occlusion governing polar localization and asymmetric segregation during vegetative growth. However, our findings also revealed that PAs can readily persist throughout the entire sporulation process after encapsulation in the forespore during sporulation. Moreover, no deleterious effects of PA presence on sporulation, germination and spore survival against heat or UV stress could be observed. Our findings therefore indicate that the sporulation process is remarkably robust against perturbations by PAs and misfolded proteins.

## 1. Introduction

Intracellular protein aggregates (PAs) tend to appear when cells encounter proteotoxic stress and accumulate denatured proteins that interact via their hydrophobic domains into macromolecular assemblies [1]. In bacteria, the cellular management of these PAs can be quite diverse. Although in the Gram-negative *Escherichia coli* model system the nucleoid-occluded polar localization of PAs forces these structures to segregate asymmetrically upon cell division [2,3,4,5,6,7,8], the larger nucleoid-to-cytoplasm ratio of *Caulobacter crescentus* seems to trap PAs within the DNA mesh at multiple locations throughout the cell volume and cause them to segregate more symmetrically [1,9,10].

In addition, the possible impact of bacterial PAs seems to be highly diverse as well. Indeed, some studies find indications of PA-borne debilitating effects ranging from cellular senescence to dormancy (e.g., persistence and the viable but not culturable (VBNC) state) and attribute these effects to a possible cytotoxicity of PAs and/or the loss of functional proteins and the squandering of cellular resources associated with protein aggregation [5,11,12,13,14,15,16]. Other studies, however, find indications of PA-mediated protective effects [2,17,18], possibly driven by the presence of chaperones induced by and/or associated with aggregating proteins.

One strategy for further assessing possible biological interferences caused by PAs is to study how well intricate cellular processes proceed in the presence of a PA. As such, we specifically wondered whether and to what extent intracellular PAs could hamper the cellular processes of sporulation and germination in *Bacillus subtilis* [19,20]. In fact, although previous research has revealed the existence of a checkpoint mechanism that delays entry into sporulation in response to chromosomal damage [21], no such mechanism seems to have been explored for sporulation during protein stress.

In this study, we, therefore, embarked upon constructing and exploring a synthetic PA model system in *B. subtilis* that allows for the inducible production of a fluorescently trackable inert aggregation-prone protein. Using this tool, we examined how robustly sporulation and germination proceed in the presence of an intracellular PA.

## 2. Materials and Methods

### 2.1. Bacterial Strains and Growth Conditions

Bacterial strains and plasmids used in this study are listed in Table 1 and Table 2, respectively. For strain construction, Lysogeny Broth (LB) according to Lennox (10 g/L tryptone (Lab M, Lancashire, UK), 5 g/L yeast extract (Oxoid, Hampshire, UK), 5 g/L NaCl) was used. BHI medium (Oxoid) supplemented with 30 mM L-valine (Fisher Scientific, Pittsburgh, PA, USA) was used for time-lapse fluorescence microscopy (TLFM) germination. TLM and 15% CDM medium for TLFM sporulation experiments were made as described by previous research [22,23], but glucose was omitted from CDM to improve sporulation efficiency. Starch agar plates (3 g/L soluble starch (Sigma-Aldrich, Saint Louis, MO, USA) and 28 g/L Nutrient Agar (Oxoid) were used to confirm double homologous recombination at the *amyE* locus. Stationary phase cultures were made by growing cultures for 15 h under well-aerated conditions (200 rpm on an orbital shaker).

Cultures of PS832 were made naturally competent by picking a colony from a fresh stock plate and growing it overnight at 30 °C in 3 mL of SP medium. The following morning, the culture was diluted 1/50 by adding 200 µL to 10 mL of fresh SP medium containing the appropriate antibiotic. The culture was incubated at 30 °C until an OD_600_ of 0.6–0.8 was reached, indicating that cells were ready to be transformed. Plasmid transformation was achieved by adding 5 µL of plasmid (concentration between 50 and 150 ng/µL) to 500 µL of cell culture. Cells were resuscitated at 30 °C for approx. 1 h in culture tubes before plating on selective media. Plates were incubated overnight at 30 °C.

Where appropriate, the following antibiotics were added to the medium at the indicated final concentrations: 100 µg/mL ampicillin (Fisher Scientific; *E. coli*), 50 µg/mL spectinomycin (Sigma-Aldrich; *B. subtilis*) and 20–1000 µM IPTG.

To create a translational fusion of *gfp(Sp)* to *cI78^EP8^* or *cI78^WT^,* linked by a GS linker (Gly-Ser-Gly-Ser-Gly-Ser), an amplicon was created on pTrc99A-*P_trc_-mCer-cI78^EP8^* or pTrc99A-*P_trc_-mCer-cI78^WT^*, respectively, with primer pair P1 and P2, and on pDR111-*gfp(Sp)* with primer pair P3 and P4 (Table 3). Both amplicons are flanked by ca. 50 bp homologous regions to facilitate Gibson assembly (New England BioLabs, Ipswich, MA, USA), which inserts *cI78^EP8^*/*cI78^WT^* downstream of *gfp(Sp)* in the pDR111-*gfp(Sp)* plasmid. The resulting plasmids were transformed into electrocompetent DH5α cells.

The *P_Hyperspank_* locus in pDR111-*gfp(Sp)*, pDR111-*gfp(Sp)-cI78^EP8^* and pDR111-*gfp(Sp)-cI78^WT^* is flanked by regions homologous to the *B. subtilis amyE* locus that allow for chromosomal integration after transformation of these suicide vectors. Plasmids were transformed to naturally competent *B. subtilis* PS832 cells and chromosomal recombination of the *P_Hyperspank_* locus was selected for on LB agar supplemented with spectinomycin. Double homologous recombination at the *amyE* locus was confirmed by lack of amylase activity after overnight growth on starch plates, revealed with lugol staining (Sigma-Aldrich).

All constructed plasmids and chromosomal insertions were initially confirmed by PCR with primer pairs attaching outside of the region of insertion (Table 3). Correct insertions were further verified by sequencing (Macrogen, Amsterdam, the Netherlands).

### 2.2. Sporulation Induction for TLFM

To induce sporulation on agarose pads for TLFM, 1 mL of stationary phase TLM cultures (grown in 20 mL at 30 °C) was diluted 1/25 in 24 mL of pre-warmed 15% CDM and grown to exponential phase by incubating for approx. 4 h at 30 °C. When the culture reached an OD_600_ of approximately 0.015, an appropriate dilution of the culture was placed on 15% CDM agarose pads and incubated during TLFM at 30 °C.

### 2.3. Spore Harvesting

Bulk spore suspensions were made by growing cells on Nutrient Agar for 7 days at 37 °C. Spores were subsequently harvested by washing 3 times with milli-Q (Millipore Simplicity Water Purification System; Merck Millipore, Burlington, MA, USA) at 4000× *g* (10 min, 4 °C) and stored at 4 °C.

### 2.4. Germination Induction for TLFM

A 20 µL spore suspension was heat activated in a heating block (70 °C, 30 min) to facilitate synchronous germination and inactivate vegetative cells. An appropriate dilution was subsequently placed on BHI agarose pads supplemented with L-valine for induction of germination at 37 °C during TLFM.

### 2.5. Semi-Lethal Treatment of Spore Suspensions

A 20 µL spore suspension was transferred aseptically to a sterile PCR tube and heat treated for 10 min in a Biometra T3000 Thermocycler (Biometra, Göttingen, Germany) at 90 °C. Alternatively, 100 µL of a spore suspension was exposed to 0.006 J of UV in a BLX-254 (Vilber Lourmat, Collégien, France) while shaking (100 rpm). Spore suspensions were subsequently monitored with TLFM on BHI agarose pads supplemented with L-valine for germination and outgrowth.

### 2.6. Time-Lapse Fluorescence Microscopy (TLFM)

Appropriate culture or spore suspension dilutions were placed on agarose pads containing the appropriate medium and supplemented with 1.5% LSL-LE 8200 agarose (Lonza, Basel, Switzerland) on a microscopy slide and covered with a cover glass attached to a 125 µL Gene Frame (Thermo Fisher Scientific, Waltham, MA, USA). TLFM was performed on a Ti-Eclipse inverted microscope (Nikon, Champigny-sur-Marne, France) equipped with a 60× Plan Apo λ oil objective, a TI-CT-E motorized condenser and a Nikon DS-Qi2 camera. GFP was imaged using a quad-edge dichroic (395/470/550/640 nm) and FITC single emission filters. A SpectraX LED illuminator (Lumencor, Beaverton, OR, USA) was used as a light source, using the 470/24 excitation filter. Temperature was controlled at 30 °C (for sporulation) or 37 °C (for germination) with an Okolab cage incubator (Okolab, Ottaviano, Italy). Images were acquired using NIS-Elements software (version 4.51, Nikon) and the resulting pictures were further handled with the open source software ImageJ (version 1.54d) [24].

### 2.7. Statistical Analysis

Statistical analyses were carried out using the open source software R (version 4.2.3, R Core Team, Vienna, Austria, 2023). Differences were regarded as significant when the *p*-value was ≤0.05. Means and the corresponding standard errors were calculated over three independent experiments.

## 3. Results

### 3.1. Implementing a Synthetic PA Model System in B. subtilis

**Figure 1:**
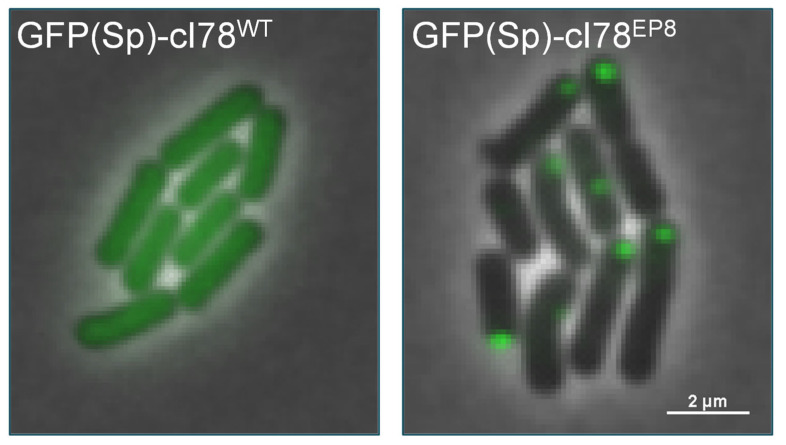
PA model system in *B. subtilis.* Representative overlay images of phase contrast and GFP fluorescence channels of *B. subtilis* PS832 *amyE::P_Hyperspank_ gfp(Sp)-cI78^WT^* (left panel) and PS832 *amyE::P_Hyperspank_ gfp(Sp)-cI78^EP8^* (right panel) microcolonies grown for 2 h on 15% CDM agarose pads supplemented with 100 µM IPTG. Scale bar corresponds to 2 µm.

In order to create a model system that allows the single-cell investigation of PA dynamics and management in *B. subtilis,* we adapted a previously established system from *E. coli*, in which a truncated version of the cI protein of phage λ was made aggregation-prone by random mutagenesis [2]. More specifically, the first 77 amino acids, entailing the N-terminal DNA-binding domain of cI, were removed to obtain cI78^WT^, after which an aggregating version of this protein was obtained via an error-prone PCR screen (i.e., cI78^EP8^; harboring 1 synonymous and 3 nonsynonymous mutations compared to cI78^WT^) [2]. Both this spontaneously aggregating version (cI78^EP8^) and its soluble control (cI78^WT^) were C-terminally fused to a monomeric GFP optimized for low-GC content Gram-positive bacteria (GFP(Sp)) and expressed from the chromosomal *amyE* locus under control of an IPTG-inducible promoter (*P_Hyperspank_*) and a strong ribosomal binding site (R0) [25]. An additional control strain that exclusively expresses GFP(Sp) from this locus was constructed as well. Inducing GFP(Sp)-cI78^EP8^ expression through IPTG exposure results in the formation of fluorescently trackable foci, whereas fluorescence in the GFP(Sp)-cI78^WT^ control is homogeneously dispersed throughout the cytoplasm (Figure 1).

### 3.2. PAs Readily Persist throughout the Sporulation and Germination Processes

**Figure 2:**
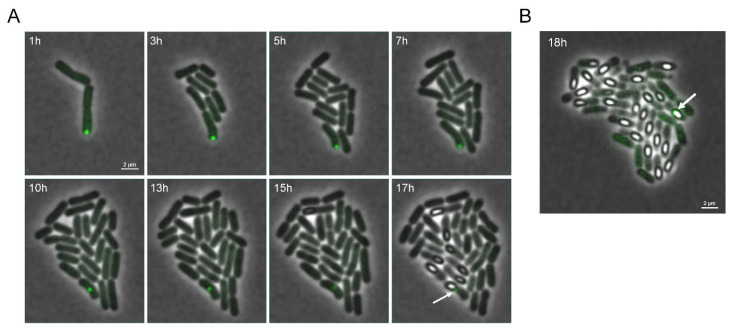
Example of PA dynamics during growth and sporulation. Representative overlay images of phase contrast and GFP fluorescence channels of a PS832 *amyE::P_Hyperspank_ gfp(Sp)-cI78^EP8^* microcolony grown on IPTG-free sporulation-inducing 15% CDM agarose pads for the indicated time points after 4 h of prior induction during liquid exponential growth with 20 µM IPTG to preload the founder cells with PAs. The white arrow in panel (**A**) indicates a fluorescent PA that remained in the mother cell, whereas the white arrow in panel (**B**) indicates a PA encapsulated in the endospore itself. Scale bars correspond to 2 µm.

**Figure 3:**
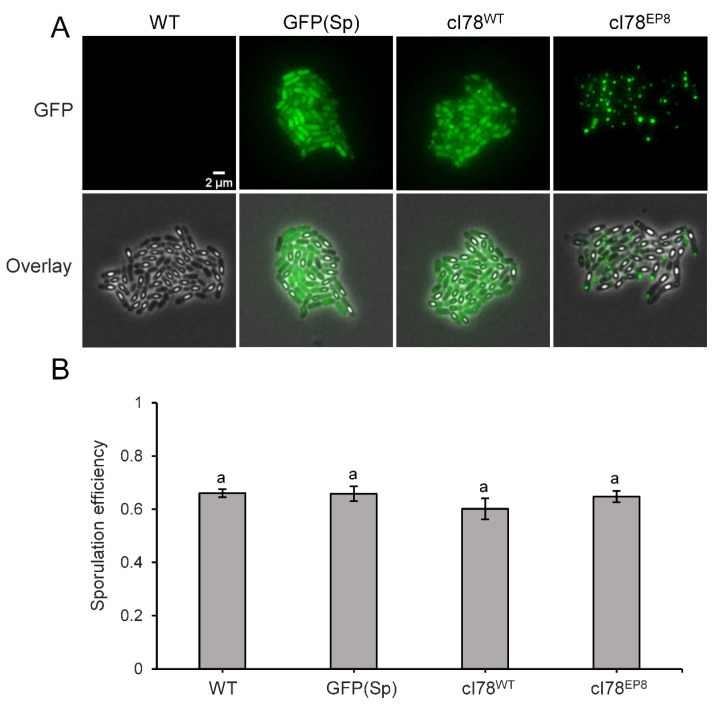
Sporulation is not perturbed by GFP(Sp)-cI78^EP8^ aggregates. (**A**) Representative GFP fluorescence and overlay images of PS832, PS832 *amyE::P_Hyperspank_ gfp(Sp)*, PS832 *amyE::P_Hyperspank_ gfp(Sp)-cI78^WT^* and PS832 *amyE::P_Hyperspank_ gfp(Sp)-cI78^EP8^* microcolonies grown on sporulation-inducing 15% CDM agarose pads supplemented with 1 mM IPTG for 20 h. Scale bar corresponds to 2 µm. (**B**) Sporulation efficiency was determined with TLFM by monitoring the fraction of PS832, PS832 *amyE::P_Hyperspank_ gfp(Sp)*, PS832 *amyE::P_Hyperspank_ gfp(Sp)-cI78^WT^* and PS832 *amyE::P_Hyperspank_ gfp(Sp)-cI78^EP8^* cells that differentiated into endospores over three independent experiments in the timeframe of the experiment (20 h). The remaining fraction either lysed or stopped growing. On average, 143 cells were observed per strain and per replicate and sample sizes were always between 108 and 168 cells. Different letters indicate statistically significant (ANOVA followed by Tukey HSD post-hoc test, *p*-value ≤ 0.05) differences among strains. Error bars indicate the standard errors.

Intracellular PA dynamics were tracked microscopically after inducing PA formation in exponential PS832 *amyE::P_Hyperspank_ gfp(Sp)-cI78^EP8^* cultures and subsequently monitoring growing cells on IPTG-free nutrient-poor agarose pads to trigger sporulation. As such, and in agreement with recent findings of Matavacas et al. (2023) [26], we first observed that during vegetative growth *B. subtilis* PAs clearly adhered to the *E. coli* paradigm of nucleoid-enforced polar localization and asymmetric segregation during vegetative growth (Figure 2). Interestingly, during subsequent sporulation, we could observe that PAs in sporulating cells became highly mobile and sampled more of the cytoplasmic environment, presumably due to an increase in nucleoid-free space as a result of the chromosome condensing into an axial filament during the early stages of sporulation [27]. Eventually, the fluorescent PA could become encapsulated in the forespore or remain in the mother cell upon sporulation (Figure 2), and we found that PA encapsulation occurred in ca 8.7% (se = 1.1%) of sporulating cells that harbor a single PA upon sporulation. Surprisingly, the sporulation process seemed unperturbed by the presence of a preformed inert PA in either the mother cell or forespore. In fact, even when severely inducing misfolded protein production (i.e., using a high IPTG concentration) during the sporulation process itself (Figure 3A), no significant decrease in sporulation efficiency was found compared to the control strains (Figure 3B).

**Figure 4:**
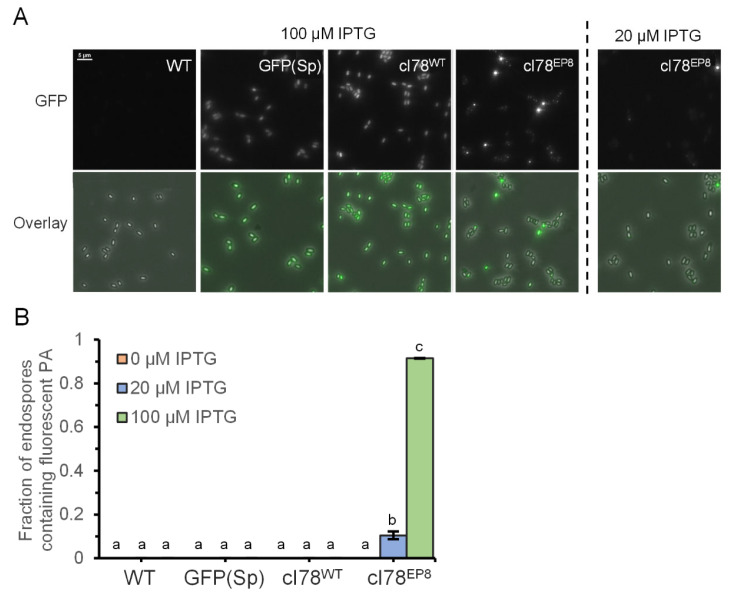
GFP(Sp)-cI78^EP8^ Pas can become encapsulated in endospores. (**A**) Representative images of GFP fluorescence (top) and the corresponding overlay images of phase contrast and GFP fluorescence channels (bottom) of PS832, PS832 *amyE::P_Hyperspank_ gfp(Sp)*, PS832 *amyE::P_Hyperspank_ gfp(Sp)-cI78^WT^* and PS832 *amyE::P_Hyperspank_ gfp(Sp)-cI78^EP8^* spore crops harvested after prior growth, starvation and sporulation of corresponding vegetative cell on Nutrient Agar plates supplemented with the indicated IPTG concentrations. Scale bar corresponds to 5 µm. (**B**) The fraction of endospores containing a fluorescent PA of PS832, PS832 *amyE::P_Hyperspank_ gfp(Sp)*, PS832 *amyE::P_Hyperspank_ gfp(Sp)-cI78^WT^* and PS832 *amyE::P_Hyperspank_ gfp(Sp)-cI78^EP8^* spore crops harvested after prior growth, starvation and sporulation of corresponding vegetative cell on Nutrient Agar plates supplemented with 0 (orange), 20 (blue) and 100 µM (green) IPTG. Error bars indicate the standard errors over three independent experiments. On average, 85.3 spores were observed per strain, replicate and condition, and sample sizes were always between 71 and 94 spores. Different letters indicate statistically significant (ANOVA followed by Tukey HSD post-hoc test, *p*-value ≤ 0.05) differences among strains and conditions.

To more systematically quantify the encapsulation of PAs in endospores, we harvested spore suspensions after starvation on solid medium in the presence of varying concentrations of IPTG and noted that ca. A total of 10.4% (se = 1.7%) of spores contained a fluorescent PA when exposed to low levels of IPTG induction during growth and sporulation, whereas no fluorescent foci could be observed in the PS832 wild type, PS832 *amyE::P_Hyperspank_ gfp(Sp)-cI78^WT^* or PS832 *amyE::P_Hyperspank_ gfp(Sp)* endospores, regardless of IPTG induction levels (Figure 4A,B). These observations again highlight the occurrence of PA encapsulation in endospores. Moreover, the fraction of PA-harboring endospores was as high as 91.4% (se = 0.1%) under high levels of IPTG induction (Figure 4A,B), which may potentially be attributed to the sporulating cell harboring multiple Pas as a result of strong induction (making it less likely for the endospore to avoid PA encapsulation) and/or additional PA formation occurring in the forespore itself (as opposed to true encapsulation of preexisting Pas).

**Figure 5:**
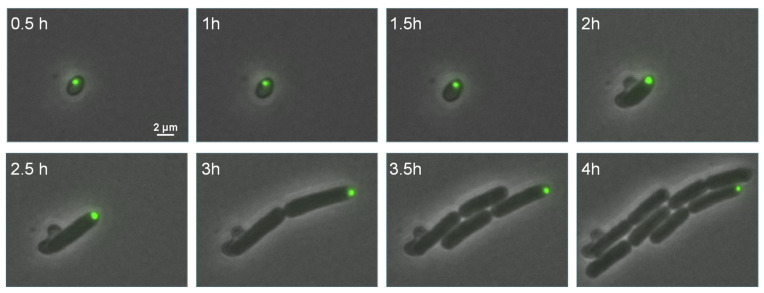
PA dynamics during germination and outgrowth. Representative overlay images of phase contrast and GFP fluorescence channels of a PS832 *amyE::P_Hyperspank_ gfp(Sp)-cI78^EP8^* PA-harboring endospore grown on germination-inducing BHI agarose pads for the indicated time points. Endospores were loaded with Pas by prior growth, starvation and sporulation of corresponding vegetative cells on Nutrient Agar plates supplemented with 20 µM IPTG. Scale bar corresponds to 2 µm.

**Figure 6:**
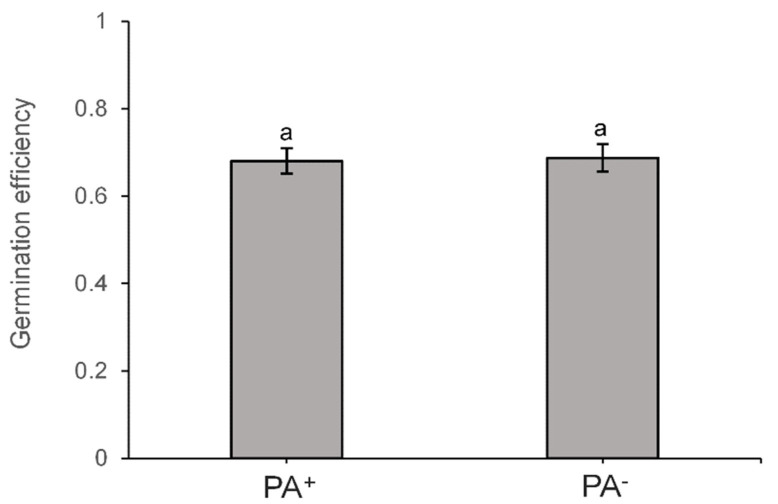
Germination efficiency is not affected by GFP(Sp)-cI78^EP8^ aggregates. Germination efficiency was determined by monitoring the fraction of germinating cells of PS832 *amyE::P_Hyperspank_ gfp(Sp)-cI78^EP8^* spores on BHI agarose pads. Spore crops were harvested after prior growth, starvation and sporulation of corresponding vegetative cells on Nutrient Agar plates supplemented with 20 µM IPTG, creating subpopulations of PA-bearing (PA^+^) and PA-free (PA^−^) endospores. On average, 94.2 spores were observed per bin and per replicate and sample sizes were always between 77 and 129 spores. Different letters indicate statistically significant (two-sided Student *t* test, *p*-value ≤ 0.05) differences among bins. Error bars indicate the standard errors over three independent experiments.

Finally, GFP(Sp)-cI78^EP8^ Pas encapsulated into endospores could readily pass the endospore stage and became asymmetrically segregated after germination and outgrowth (Figure 5). In fact, germination efficiency did not become impeded in endospores harboring a GFP(Sp)-cI78^EP8^ PA compared to PA-free endospores (Figure 6).

### 3.3. PA-Harboring Endospores Are Not Affected in Heat or UV Survival

**Figure 7:**
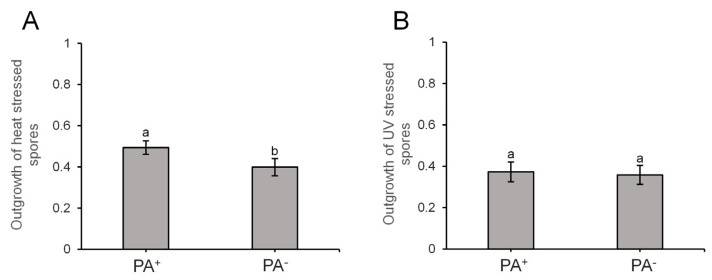
GFP(Sp)-cI78^EP8^ aggregates do not impede heat or UV survival of endospores. Outgrowth was determined by monitoring the fraction of germinating and dividing cells of stress-exposed PS832 *amyE::P_Hyperspank_ gfp(Sp)-cI78^EP8^* spores on BHI agarose pads. Spore crops were harvested after starvation on Nutrient Agar plates supplemented with 20 µM IPTG, creating subpopulations of PA-bearing (PA^+^) and PA-free (PA^−^) endospores. (**A**) Outgrowth of endospores exposed to heat (90 °C for 10 min) determined over 6 independent experiments. On average, 100.2 spores were observed per bin and per replicate and sample sizes were always between 71 and 136 spores. (**B**) Outgrowth of endospores exposed to UV (0.006 J) determined over 3 independent experiments. On average, 65.5 spores were observed per bin and per replicate and sample sizes were always between 39 and 90 spores. For panels A and B, different letters indicate statistically significant (two-sided paired Student *t* test, *p*-value ≤ 0.05) differences among strains. Error bars indicate the standard errors.

Since GFP(Sp)-cI78^EP8^ Pas thus appear to be readily encapsulated in the forespore without influencing further sporulation or subsequent germination, we wondered whether the presence of these structures could somehow affect spore survival after exposure to stressful conditions. To this end, we harvested isogenic spore crops containing both PA-bearing and PA-free endospores and challenged them to semi-lethal doses of heat (Figure 7A) or UV (Figure 7B). However, endospores harboring Pas did not become impeded in their ability to survive semi-lethal exposure to either of these stresses (Figure 7). In fact, Pas may even confer a slight benefit to survival in heat-exposed spores (Figure 7A), although this effect was small and barely significant (*p*-value = 0.04).

## 4. Discussion

In this study, we developed a novel PA model system to investigate the intracellular PA dynamics in *B. subtilis*. The system allows for the inducible production of synthetic fluorescently trackable PAs (GFP(Sp)-cI78^EP8^), and provides suitable control strains that produce soluble variants (GFP(Sp)-cI78^WT^ and GFP(Sp)). Previous attempts at making fluorescent PA reporters in *B. subtilis* have mainly involved fluorescently tagging components of the proteostatic network that are thought to colocalize to PAs (e.g., the protease ClpP and its unfoldases ClpC, ClpX and ClpE) [28,29,30,31]. However, in the light of later research that showed that many fluorescent fusion proteins commonly used to study cellular localization have an innate tendency to trivially assemble in foci [32], it remains unclear whether these fluorescent PA reporters represent the native behavior of the proteostatic components and/or PAs. Additionally, these proteostasis components have known roles in important cellular functions such as sporulation [28,31,33,34,35,36,37,38] and competence [39], which could be perturbed when these proteins are fluorescently tagged without adequate validation. Fluorescently labeled malate dehydrogenase (Mdh) has also been utilized to visualize subcellular PAs because Mdh is known to unfold and co-aggregate with PAs upon heat stress [40,41]. Additionally, a recent study revealed that xylose-mediated induction of a fluorescently tagged sHSP, YocM, leads to fluorescently trackable colocalization to salt and heat stress-induced PAs [41]. Although YocM-mCherry did not seem to have retained the full YocM activity, it provides yet another intriguing possibility for the investigation of PAs in *B. subtilis.*

Using the GFP(Sp)-cI78^EP8^ model system, we could investigate the interplay between synthetic PAs and sporulation/germination processes. Our results reveal a remarkable robustness of endospores (and the process of sporulation itself) against proteotoxic stress and PAs. Indeed, we could not find any PA-mediated perturbance on sporulation, germination, or on the survival capabilities of spores against heat or UV stress. We even noticed that GFP(Sp)-cI78^EP8^ PAs can be readily encapsulated in endospores. These results do not seem to indicate the existence of a protein stress checkpoint mechanism during sporulation or any negative effect on sporulation mediated by the production of a (synthetic) misfolded protein. However, it remains to be investigated whether these observations can be extrapolated to naturally occurring (e.g., stress-induced) misfolded proteins and PAs because the sequestering of functional cellular proteins into PAs and their potential subsequent release during disaggregation may more significantly impact the phenotypic output of the cell.

The study and comparison of a variety of bacterial model organisms and processes will be crucial to develop in-depth and generally applicable insights about PA management and its phenotypic consequences in bacteria. As such, our study provides a route to developing this understanding of the important Gram-positive *B. subtilis* model organism and contributes to the tools to facilitate further investigations in this field.

## Figures and Tables

**Table 1:** Overview of strains used in this study.

Strain	Description	Source or Reference
*Escherichia coli*		
DH5α	Used for transformation of plasmids	Laboratory collection
*Bacillus subtilis*		
PS832	*B. subtilis* PS832 wild type; prototrophic derivative of *B. subtilis* 168	Received from Peter Setlow (University of Connecticut)
PS832 *amyE*::*P_Hyperspank_ gfp(Sp)-cI78^EP8^*	PS832 with *P_Hyperspank_ gfp(Sp)-cI78^EP8^* cassette inserted in its *amyE* locus	This study
PS832 *amyE*::*P_Hyperspank_ gfp(Sp)-cI78^WT^*	PS832 with *P_Hyperspank_ gfp(Sp)-cI78^WT^* cassette inserted in its *amyE* locus	This study
PS832 *amyE*::*P_Hyperspank_ gfp(Sp)*	PS832 with *P_Hyperspank_ gfp(Sp)* cassette inserted in its *amyE* locus	This study

**Table 2:** Overview of plasmids used in this study.

Plasmid	Description	Source or Reference
pTrc99A-*P_trc_-mCer-cI78^EP8^*	Contains *cI78^EP8^* which encodes an aggregate-prone truncated version of the lambda prophage repressor protein cI	[1]
pTrc99A-*P_trc_-mCer-cI78^WT^*	Contains *cI78^WT^* which encodes a soluble truncated version of the lambda prophage repressor protein cI	[1]
pDR111-*gfp(Sp)*	Contains *gfp(Sp)* under IPTG-inducible *P_Hyperspank_* to integrate into the *amyE* locus	[20]
pDR111-*gfp(Sp)-cI78^EP8^*	Contains *gfp(Sp)-cI78^EP8^* under IPTG-inducible *P_Hyperspank_* to integrate into the *amyE* locus	This study
pDR111-*gfp(Sp)-cI78^WT^*	Contains *gfp(Sp)-cI78^WT^* under IPTG-inducible *P_Hyperspank_* to integrate into the *amyE* locus	This study

**Table 3:** Overview of primers used in this study. When relevant, primer attachment sites are indicated in bold.

Name	Sequence	Purpose
P1	ACACATGGTATGGATGAATTGTATAAAGGCTCTGGCTCTGGCTCT**AGCCCTTCAATCGCCAGAGAA**	Creates an amplicon of *cI78^EP8^* and *cI78^WT^* to insert into pDR111-*gfp(Sp)*
P2	CAGAATTGCCGACCTTGACTAGTGCTCATTATTA**GCCAAACGTCTCTTCAGGCC**	Creates an amplicon of *cI78^EP8^* and *cI78^WT^* to insert into pDR111-*gfp(Sp)*
P3	AGAGCCAGAGCCAGAGCC**TTTATACAATTCATCCATACCATGTGT**	Linearizes the pDR111-*gfp(Sp)* vector to insert the *cI78^EP8^* and *cI78^WT^* amplicon
P4	TAATAATGAGCACTAGTCAAGGTCG	Linearizes the pDR111-*gfp(Sp)* vector to insert the *cI78^EP8^* and *cI78^WT^* amplicon
P5	CGTTTCGGTGATGAAGATCTTC	Control and sequencing of insertions in pDR111-*gfp(Sp)*
P6	CCTCGTTTCCACCGAATTAGC	Control and sequencing of insertions in pDR111-*gfp(Sp)*
P7	GTTCTGTTTCTGCTTCGGTATG	Control and sequencing of insertions in the *amyE* locus
P8	GCAAATGCATAACTGCTTCCAAC	Control and sequencing of insertions in the *amyE* locus

## Data Availability

Data available upon request.
